# No evidence of a death-like function for species B1 human adenovirus type 3 E3-9K during A549 cell line infection

**DOI:** 10.1186/1756-0500-5-429

**Published:** 2012-08-11

**Authors:** Kathryn M Frietze, Samuel K Campos, Adriana E Kajon

**Affiliations:** 1 Infectious Disease Program, Lovelace Respiratory Research Institute, 2425 Ridgecrest Drive SE, Albuquerque, NM, USA; 2 Department of Immunobiology, BIO5 Institute, University of Arizona, Keating Building Rm 423, 1657 E. Helen St, Tucson, 85721-0240, AZ, USA; 3 Department of Molecular Genetics and Microbiology, University of New Mexico, Albuquerque, NM, 87131-0001, USA

**Keywords:** Adenovirus, E3 region, Genetic polymorphism, Virus egress

## Abstract

**Background:**

Subspecies B1 human adenoviruses (HAdV-B1) are prevalent respiratory pathogens. Compared to their species C (HAdV-C) counterparts, relatively little work has been devoted to the characterization of their unique molecular biology. The early region 3 (E3) transcription unit is an interesting target for future efforts because of its species-specific diversity in genetic content among adenoviruses. This diversity is particularly significant for the subset of E3-encoded products that are membrane glycoproteins and may account for the distinct pathobiology of the different human adenovirus species. In order to understand the role of HAdV-B-specific genes in viral pathogenesis, we initiated the characterization of unique E3 genes. As a continuation of our efforts to define the function encoded in the highly polymorphic ORF E3-10.9K and testing the hypothesis that the E3-10.9K protein orthologs with a hydrophobic domain contribute to the efficient release of viral progeny, we generated HAdV-3 mutant viruses unable to express E3-10.9K ortholog E3-9K and examined their ability to grow, disseminate, and egress in cell culture.

**Results:**

No differences were observed in the kinetics of infected cell death, and virus progeny release or in the plaque size and dissemination phenotypes between cells infected with HAdV-3 E3-9K mutants or the parental virus. The ectopic expression of E3-10.9K orthologs with a hydrophobic domain did not compromise cell viability.

**Conclusions:**

Our data show that despite the remarkable similarities with HAdV-C E3-11.6K, HAdV-B1 ORF E3-10.9K does not encode a product with a “death-like” biological activity.

## Background

Subspecies B1 human adenoviruses (HAdV-B1) are important causes of acute respiratory disease in children and military recruits [1-6]. In particular, serotypes 3 and 7 (HAdV-3 and HAdV-7) and their genomic variants are commonly isolated in association with severe pediatric acute respiratory disease worldwide [7-10]. Compared to their species C (HAdV-C) counterparts, relatively little work has focused specifically on characterizing the unique molecular biology of these important human pathogens. The early region 3 (E3) transcription unit is of particular interest for its species-specific diversity in genetic content among adenoviruses [11,12] and for encoding important modulators of the host response to infection [13-16]. This diversity is particularly significant for the subset of E3-encoded products that are membrane glycoproteins and may account for the distinct pathobiology of the different human adenovirus species. Between the highly conserved E3-19K and RIDα, HAdV-A to -F encode unique repertoires of genes whose products are membrane proteins that belong to the CR1 protein superfamily (pfam02440 in the NCBI CDD) and that are likely to be responsible for their distinct pathobiology. Adenovirus CR1 genes are designated CR1α, β, γ and δ based on their order in the E3 cassette and exhibit some homology with the highly diverse human cytomegalovirus RL11 gene family [17]. With the exception of HAdV-C E3-11.6K aka adenovirus death protein (ADP) and E3-6.7K [18-20] no functional role has been identified for adenovirus (or cytomegalovirus) -encoded CR1 proteins.

We previously reported the initial characterization of the uniquely diverse family of orthologs encoded by HAdV-B1s in ORF E3-10.9K/CR1δ [21,22]. This polymorphic HAdV-B1-specific E3 ORF encodes proteins ranging in predicted size from 4.8 kDa to 10.9 kDa, depending on the serotype and genomic variant [22]. Our studies showed that orthologs E3-7.7K, E3-9K, and E3-10.9K containing predicted transmembrane domains localized to the plasma membrane and to an intracellular compartment that could not be identified when expressed ectopically as EGFP-fusion proteins, while the 4.8 kDa ortholog lacking a hydrophobic domain displayed diffuse cellular localization [21]. The location of ORF E3-10.9K in the E3 transcription unit is analogous to that of HAdV-C E3-11.6K/adenovirus death protein (ADP) [11]. Like HAdV-C E3-11.6K/ADP, E3-10.9K is expressed at late time points post-infection from the adenovirus major late promoter, and exhibits similar structural features including a hydrophobic domain, molecular weight, and N- and O-linked glycosylation [21]. However, the E3-11.6K/ADP primary localization to the nuclear envelope in infected cells [23] was not observed for any of the ectopically expressed E3-10.9K ortholog fusion proteins with a hydrophobic domain [21]. This led us to hypothesize that HAdV-B1 E3-10.9Kis a homolog of HAdV-C E3-11.6K/ADP that facilitates efficient progeny virus release through a different mechanism.

In this body of experimental work, we tested this hypothesis using HAdV-3 mutant viruses lacking the ability to express ortholog E3-9K and examined their ability to egress, kill, and disseminate in cultured cells in comparison with the parental virus.

## Results

### Generation of HAdV-3-E3-9K knock-out mutants

**Figure 1:**
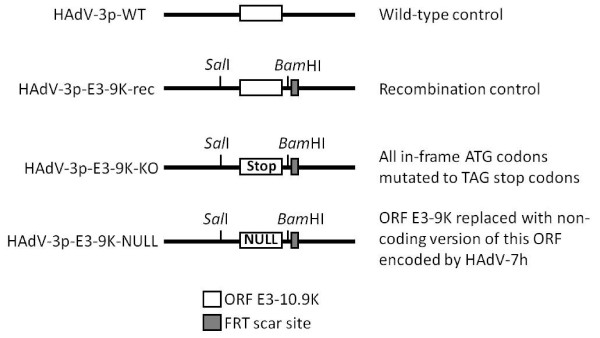
** Schematic of HAdV-3p E3-9K mutant virus genotypes.** Mutant viruses were generated by recombination of bacmid pKSB2Ad3wt with pE3-9K shuttle plasmids carrying the desired mutations of ORF E3-9K, digestion of bacmid with *Mlu*I to release the mutated HAdV-3 genome, transfection into A549 cells, and propagation of resultant mutant virus as described in the Materials and Methods section. Recombinants contain *Sal*I and *Bam*HI restriction enzyme sites and a flippase recognition target (FRT) scar site. The HAdV-3p-WT control virus was generated by linearization of pKSB2Ad3wt by digestion with *Mlu*I without prior recombination or mutation. HAdV-3p-E-3-9K-rec, the recombination control virus, was generated by recombination of pKSB2Ad3wt with unaltered pE3-9K shuttle plasmid. An E3-9K knock out mutant, HAdV-3p-E3-9K-KO, was generated by recombination of pKSB2Ad3wt with a pE3-9K shuttle plasmid that contained TAG stop codons in place of all the in-frame ATG codons present in ORF E3-9K. HAdV-3p-E3-9K-NULL was generated by recombination of pKSB2Ad3wt with a pE3-9K shuttle plasmid carrying the HAdV-7h-encoded null version of this ORF (GenBank accession # Z48954 and AF321310).

In order to investigate the role of ORF E3-10.9K in the growth and progeny release of HAdV-B1s, we generated HAdV-3p mutant viruses lacking the ability to express a protein from ORF E3-9K. HAdV-3p encodes a 9 kDa ortholog in this ORF that is expressed at early times post infection but also late from a major late promoter transcript [21]. Using recombination-based engineering of the HAdV-3p genome, we generated two control viruses (HAdV-3p-WT and HAdV-3p-E3-9Krec) and two viruses unable to express a protein from ORF E3-9K (HAdV-3p-E3-9K-KO and HAdV-3p-E3-9K-NULL). A schematic showing the HAdV-3p mutant viruses generated for these experiments is shown in Figure 1. HAdV-3p-WT was generated from bacmid pKBS2Ad3wt [24] and had no alterations to the E3 transcription unit. HAdV-3p-E3-9K-rec is a recombination control virus containing *Sal*I and *Bam*HI restriction sites flanking ORF E3-9K and a flippase recognition target (FRT) scar site. Mutant HAdV-3p-E3-9K-KO was generated by mutating all in-frame ATG codons in ORF E3-9K to TAG stop codons. Mutant HAdV-3p-E3-9K-NULL contains the non-coding version of ORF E3-10.9K encoded in the genome of HAdV-7h [22,25] and represents a naturally occurring null mutation in this ORF (Figure 1). The correct genetic make-up of all viruses was confirmed by sequencing the relevant portion of E3 region of recombinant viral DNA and examining the presence of the desired mutations, the FRT scar site, and intact neighboring ORFs (Genbank accession numbers JQ278022, JQ278023, and JQ278024). The absence of expression of ortholog E3-9 K from the KO and NULL mutants could not be verified due to the lack of a suitable antibody. 

### The absence of E3-9K does not alter HAdV-3 cytopathic effect in infected cells

**Figure 2:**
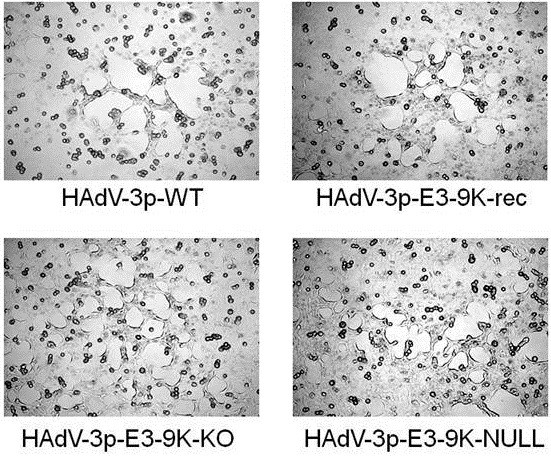
** HAdV-3p E3-9K mutant virus-induced cytopathic effect in A549 cells.** A549 cells were infected with HAdV-3 control viruses (HAdV-3p-WT, HAdV-3p-E3-9K-rec) or HAdV-3p-E3-9K mutant viruses (HAdV-3p-E3-9K-KO, HAdV-3p-E3-9K-NULL) at a MOI of 1 PFU/cell and analyzed by light microscopy 4 days pi. Classical adenovirus CPE was characterized by “webbing” or “lacy” appearance of cells followed by rounding and detaching of cells from the culture plate surface.

As an initial assessment of HAdV-3 ORF E3-9K mutant virus growth phenotype, we examined the cytopathic effect (CPE) resulting from mutant virus infection of A549 cell monolayers in comparison to the parental and recombination control viruses. A549 cells were infected at a MOI of 1 PFU/cell and analyzed by light microscopy at 4 days pi. Characteristic HAdV CPE was observed for all viruses. No difference in the progression or appearance of CPE was observed for either HAdV-3p-E3-9K-KO or HAdV-3p-E3-9K-NULL compared to controls (Figure 2).

### E3-9K mutant viruses do not have a distinct plaque phenotype compared to the parental virus

**Figure 3:**
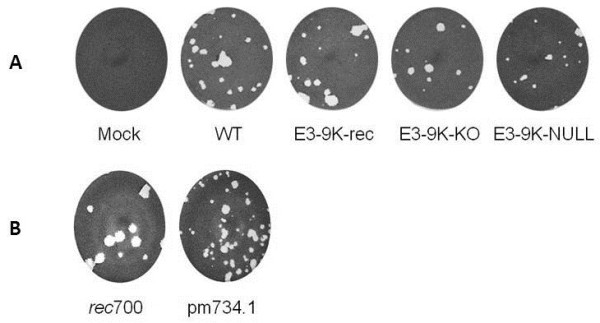
** Growth phenotype of HAdV-3p E3-9K mutant viruses in A549 cell monolayers.** A549 cells in 6-well plates were infected with approximately 20 PFU per well of HAdV-3p E3-9K mutant viruses (**A**) or HAdV-C *rec*700 and pm734.1 as a positive control (B) and overlayed with a semi-solid agarose/medium overlay. At 13 days pi (A) or 6 days pi (**B**), cells were fixed and stained with crystal violet to visualize plaques.

In order to investigate whether HAdV-3 E3-9K plays a role in plaque formation, we examined plaque size phenotype on A549 cell monolayers infected with our panel of HAdV-3-E3-9K mutant viruses. Cells were infected with approximately 20 PFU under a semi-solid agarose/medium overlay and fixed and stained at 13 days pi to visualize plaques. No difference in the sizes and morphology of plaques was observed between control and mutant HAdV-3 viruses (Figure 3A). In contrast, and as previously shown, the HAdV-C ADP knock-out mutant pm734.1 showed distinct differences in plaque size compared to parental virus *rec*700 (Figure 3B; [26]). Interestingly, plaques formed by mutant or WT HAdV-3 viruses exhibited diameters comparable to those formed by the HAdV-C ADP KO virus. 

### HAdV-3 mutant viruses lacking E3-9K do not exhibit an altered viral spread phenotype in cell culture

**Figure 4:**
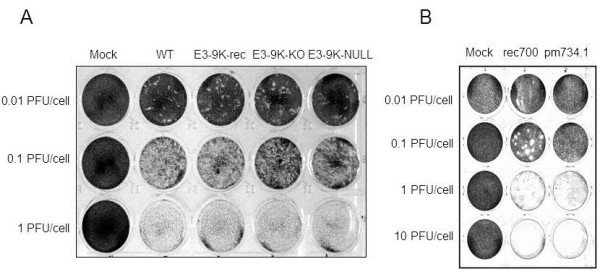
** Dissemination of HAdV-3p E3-9K mutant viruses in A549 cell monolayers.** A549 cells were infected at a MOI of 1, 0.1, or 0.01 PFU/cell with HAdV-3p E3-9K mutant viruses (**A**) or HAdV-C viruses *rec*700 and pm734.1 (**B**), replenished with liquid medium, fixed at 5 days pi, and stained with crystal violet to visualize virus-induced cell monolayer destruction.

The ability of HAdV-3-E3-9K mutant viruses to spread in cell culture was assessed by dissemination assays. In contrast to a plaque assay, a dissemination assay does not have a semi-solid agarose/medium overlay. This allows virus to freely disperse in the culture medium, instead of being confined to access neighboring cells only. HAdV-C mutant viruses without E3-11.6K/ADP show a decreased ability to disseminate in cell culture as compared to HAdV C viruses that do express E3-11.6K/ADP [27]. A549 cells were infected with HAdV-3p-E3-9K mutant viruses at MOIs of 1, 0.1, and 0.01 PFU/cell, replenished with infection medium, and fixed and stained at 5 days pi to visualize the dissemination of CPE. No difference was observed among the control HAdV-3p-WT, the recombination control HAdV-3p-E3-9K-rec, HAdV-3p-E3-9K-KO, or HAdV-3p-E3-9K-NULL (Figure 4A). In contrast and consistent with the results reported by Doronin and colleagues [27], the ADP knockout mutant pm734.1 showed distinct differences in dissemination in cell culture when compared to *rec*700 (Figure 4B). 

### The lack of E3-9K does not delay viral progeny release during HAdV-3 infection

**Figure 5:**
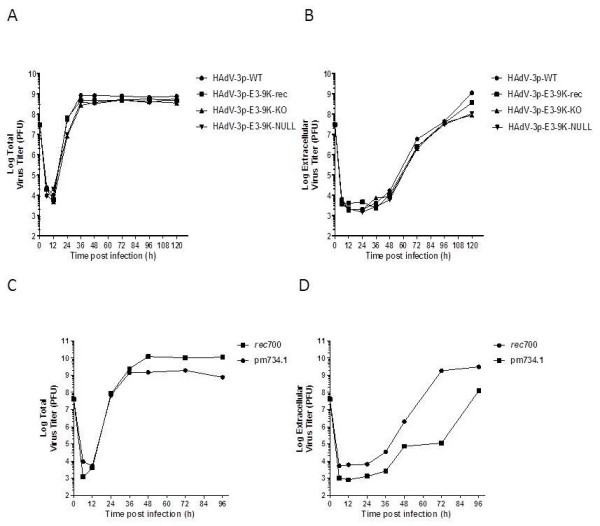
** HAdV-3p E3-9K mutant virus egress from A549 cells.** A549 cells were infected with HAdV-3p E3-9K mutant viruses (**A**-**B**) or HAdV-C *rec*700 and pm734.1 (**C**-**D**) at a MOI of 10 PFU/cell, replenished with liquid medium, and total (A,C) or extracellular (B,D) infectious virus yields were determined by plaque assay at the indicated times pi. A-B is representative of two independent experiments.

Because no differences were found in the cytopathic effect, dissemination, or plaque size phenotypes of HAdV-3p-E3-9K-KO or HAdV-3p-E3-9K-NULL compared with control viruses, we next investigated whether E3-9K played a role in virus progeny egress from infected A549 cells. Previous work on HAdV-C E3-11.6K/ADP showed a significant decrease in the progression of progeny virus egress from cells infected with mutant viruses lacking the E3-11.6K/ADP [28]. Since we hypothesized that orthologs of ORF E3-10.9K were likely the HAdV-B1 homologs of HAdV-C E3-11.6K/ADP, we carried out a virus egress experiment similar to those carried out by Tollefson and colleagues [28]. Cells were infected at a MOI of 10 PFU/cell and extracellular (supernatant) or total (cells and supernatant) infectious virus yields were determined by plaque assay. In two independent experiments, no difference was observed between the control and mutant viruses in either total or extracellular infectious virus yields (Figure 5A,B). However, a control experiment using *rec*700 and its corresponding ADP knock-out mutant pm734.1 showed obvious differences in the kinetics of virus egress, confirming the previously reported role of ADP in facilitating viral progeny release (Figure 5C,D; [28]). 

### E3-9K is not involved in progression of host cell death during HAdV-3 infection

**Figure 6:**
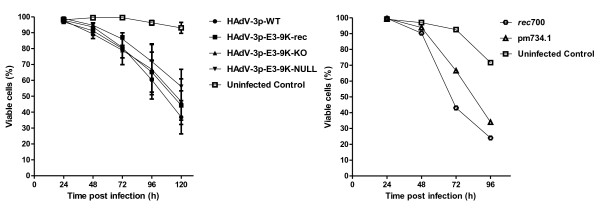
** Viability of HAdV-3p E3-9K mutant virus-infected A549 cells.A**. A549 cells were infected with HAdV-3p E3-9K mutant viruses at a MOI of 10 PFU/cell. Medium and trypsinized cells were pooled at indicated times pi, stained with an equal volume of 0.4% trypan blue solution, and approximately 200 cells were counted using a light microscope and hemocytometer. The percentage of viable cells was determined by dividing the number of unstained cells by the total number of cells counted. Error bars indicate standard error of the mean for three independent experiments. Mock-infected cells were used as a negative control. **B.** HAdV-C *rec*700 and pm734.1 controls. Representative trypan blue exclusion assay results consistent with those reported by Tollefson et al. [26] for the same viruses.

A role in the progression of host cell death has been reported for HAdV-C E3-11.6K/ADP [26,27]. In order to determine whether the HAdV-B1 ORF E3-10.9 K/CR1δ plays a role in cell killing, we used our HAdV-3-E3-9K mutant viruses to infect A549 cells at an MOI of 10 PFU/cell and examined cell viability by trypan blue exclusion assay. No difference was observed for the HAdV-3p-E3-9K-NULL or HAdV-3p-E3-9K-KO compared to control HAdV-3p-WT or recombination control HAdV-3p-E3-9K-rec (Figure 6). In contrast, ADP knock out mutant pm734.1-infected cells remained viable much longer than those infected with the parental virus control *rec*700 as originally described by Tollefson and colleagues [26]. 

### Overexpression of C-term EGFP tagged ORF E3-10.9K protein orthologs does not result in cell death

To further investigate whether expression of the E3-10.9K protein orthologs with a transmembrane domain would compromise cell viability, HeLa TREx cells overexpressing C-term EGFP fusions of E3-10.9K, and E3-9K, under tetracycline regulation (described in [21]) were examined at 48 h post induction with tetracycline using a trypan blue exclusion assay. Prior to trypan blue staining, cells were examined by bright field and fluorescence microscopy to confirm expression of the EGFP fusion proteins (Additional file 1: Figure S1). No change in permeability of HeLa TREx cells to trypan blue was observed, indicating that neither of these proteins by themselves are able induce cell death (Additional file 2: Table S1).

## Discussion

E3-11.6K/ADP, the only CR1 gene with a known function encoded in the HAdV-C genome between E3-19K and RIDα, is expressed at late stages of infection and facilitates the efficient release of virus progeny at the end of the virus life-cycle through an unknown mechanism [28]. Because of similarities in location in the E3 transcription unit, molecular weight, expression kinetics and predicted structural features, we hypothesized that HAdV-B1 E3-10.9K was a homolog of HAdV-C E3-11.6K/ADP [21,22]. The results of our experiments examining growth, dissemination and cell killing phenotypes of HAdV-3-E3-9K mutants provide evidence that this HAdV-B1-specific E3 protein does not function in an analogous manner to the well-characterized ADP. Using our HAdV-3-E3-9 K mutant viruses carrying non-coding versions of the gene we demonstrated that, in contrast to the HAdV-Cs used in this study as a reference for comparison, ortholog E3-9 K does not contribute to HAdV-3-induced cytopathic effect (Figure 2), plaque phenotype (Figure 3), dissemination in cell culture (Figure 4), kinetics of virus progeny release from infected cells (Figure 5), or host cell death (Figure 6). In addition, the over-expression as EGFP-fusion proteins of neither E3-9 K nor the longest ortholog E3-10.9 K resulted in cell death (Additional file 2: Table S1, Additional file 1: Figure S1). We previously reported that ORF E3-10.9 K orthologs with a transmembrane domain ectopically expressed as C-terminus EGFP-fusion proteins did not localize to the nuclear envelope like HAdV-C E3-11.6 K/ADP. Taken together, these data support the conclusion that the HAdV-B1 E3-10.9 K orthologs are not death-like proteins.

The lack of information on the functional role of the diverse repertoire of species-specific E3 membrane proteins remains a major limitation for the understanding of the molecular bases of the species-specific HAdV pathobiology. It is extremely likely that adenoviruses of different species differ in their life strategies and that not all of them have a need for a lytic/death-like protein at the end of their life cycle. Although ADP is important for the efficient release of HAdV-C progeny virions, it is certainly not essential. Tollefson and colleagues reported that HAdV-2/5 viruses lacking E3-11.6 K/ADP had a delayed release of virus progeny, but eventually reached virus titers equivalent to those of wild-type virus [28]. The need for a lytic protein may be linked to the ability of the virus to establish latent infections in lymphoid tissue. To the present, latency in lymphoid tissue has only been demonstrated for HAdV-C [29-31] but the role of ADP in reactivation has not been examined.

The genetic determinants and molecular mechanisms of viral progeny release have not been identified for adenoviruses other than HAdV-C. And even for the HAdV-C, other mechanisms of release of progeny virions have been suggested [32-34]. The evolutionary advantages/disadvantages of utilizing non-lytic mechanisms for viral progeny release have been discussed in the context of the antibody response from mathematical modeling of reproductive strategies [35]. It is possible that HAdVs of different species have evolved unique life strategies in response to tissue/organ specific host immune responses but the absence of a robust animal model to study HAdV pathogenesis is still a major limitation for the experimental exploration of this topic. Interestingly, in cancer animal models, Hemmiki and colleagues [36] showed that wt HAdV-3 has an oncolytic activity comparable to that of wt HAdV-5.

## Conclusions

Our data show that despite the remarkable similarities with HAdV-C E3-11.6K, HAdV-B1 E3-10.9K does not encode a product with a “death-like” biological activity. Recent molecular epidemiology studies have shown that the most prevalent strains of HAdV-B1 isolated from cases of acute respiratory disease encode versions of ORF E3-10.9K that contain truncating or null mutations [10,37,38] suggesting the existence of a selective advantage for loss of this gene among pathogenic HAdV-B1s. Interestingly, the genomes of HAdV-B2s appear to be naturally occurring deletion mutants for this ORF [39-41]. The investigation of the role of the HAdV-B-specific E3 CR1 genes, E3-20.1K and E3-20.5K in virus growth and progeny release is currently in progress in our laboratory.

Like the majority of the E3 genes with a known function, E3-10.9K may be involved in the modulation of some aspect of the host response to infection. The presence of a putative tyrosine sorting motif in the C terminus of E3-9K and E3-10.9K [21] suggest that these orthologs may exert their function by exploitation of intracellular trafficking pathways like the RID complex proteins [42]. Exploratory work investigating protein interactions with cellular and/or other viral proteins will likely be one of the few available options to obtain valuable clues to the function and possible mechanism of action of HAdV-B E3-10.9K and other adenovirus E3-encoded proteins.

## Methods

### Cells, viruses, media, and growth conditions

A549 cells (ATCC #CCL-185) were grown in 8% (v/v) newborn calf serum-supplemented Eagle Minimum Essential Medium (EMEM) (A549 Growth Medium). HAdV-infected cells were maintained in 2% (v/v) new born calf serum-supplemented EMEM (A549 Infection Medium). Plaque assays were overlayed with 2% (v/v) new born calf serum-supplemented EMEM with 0.7% (w/v) low melt agarose (A549 Overlay Medium). The HAdV-C viruses *rec*700 and pm734.1 were obtained from Dr. William Wold, Saint Louis University and used as controls in our experiments. The *rec*700 virus, an Ad2-Ad5-Ad2 recombinant [43], was used in our experiments as the wild type parental virus control. The pm734.1 virus is an E3-11.6K/ADP knock-out mutant derived from *rec*700 [26,28]. Virus stocks for all experiments described here were grown and their titers determined by standard plaque assay in A549 cell monolayers.

### Generation of HAdV-3 mutant viruses

To generate HAdV-3 mutant viruses, we utilized a recombination-based approach [21]. Briefly, the highly efficient bacteriophage λ*Red* recombination system [44] was used to generate HAdV-3 clones encoding two non-coding versions of ORF E3-9K: HAdV-3-E3-9K-KO and HAdV-3-E3-9K-NULL. Bacmid pKSB2Ad3wt, which contains the full-length genome of HAdV-3 prototype strain GB (HAdV-3p), was a gift from Dr. Silvio Hemmi [24]. Using the previously described pE3-9K shuttle plasmid [21], a series of mutations to ORF E3-9K were introduced. To generate HAdV-3-E3-9K-KO, site-directed mutagenesis was carried out to change all in-frame Met codons (ATG) to stop codons (TAG). To generate HAdV-3-E3-9K-NULL, ORF E3-9K was replaced by the non-coding version of ORF E3-10.9K encoded by HAdV-7 h strain Argentina 87–922 [22,25]. The shuttle plasmids carrying the desired mutations of ORF E3-9K were then recombined with pKSB2Ad3wt to generate bacmids harboring the mutant HAdV-3 genomes. The new bacmids were digested with *Mlu*I to release the mutated HAdV genome, and transfected into A549 cells for mutant virus isolation and propagation as previously described [21]. Mutant viruses were quality controlled by sequencing of the portion of the E3 transcription unit comprising the mutated sites and the flanking ORFs E3-20.5 K and RIDα (Genbank accession numbers JQ278022, JQ278023, and JQ278024) and restriction enzyme analysis of genomic DNA with *Bam*HI and *Sal*I (data not shown).

### Virus dissemination assays

A549 cells plated on 24-well culture plates were infected at a MOI of 1, 0.1, or 0.01 PFU/cell with each virus. After incubation for 5 days, medium was aspirated and cells were fixed in 1% formaldehyde and stained with Accustain crystal violet solution (Sigma-Aldrich, St. Louis, MO).

### Virus plaque size assays

A549 cells plated on 6-well culture plates were infected with approximately 20 PFU of virus per well. After adsorption for 1 hour at 37°C with periodic rocking to distribute inoculum, cells were covered with A549 Overlay Medium (described above). Plates were incubated for 13 days (HAdV-3 viruses) or 6 days (HAdV-C viruses) and then fixed in 1% formaldehyde and stained with Accustain crystal violet solution (Sigma-Aldrich, St. Louis, MO).

### Virus egress assays

A549 cells plated on 60 mm dishes were infected at a MOI of 10 PFU/cell. After adsorption for 1 hour at 37°C, cells were washed 3 times with PBS to remove excess extracellular virus. At 6, 12, 24, 36, 48, 72, 96, and 120 h pi, extracellular and total virus samples were harvested. In order to collect total virus samples, infected cells and supernatant were collected and freeze-thawed 3 times at −80°C and room temperature. Samples were centrifuged at 300 x g for 5 min to remove cellular debris, and the supernatant was collected. In order to collect extracellular virus samples, the supernatant from infected cells was collected and transferred to a 5 mL round-bottomed culture tube. Cells and debris were removed by centrifugation at 300 x g for 5 min, and the supernatant was collected. Infectious virus titers in all samples were determined by plaque assay on A549 cells.

### Infected cell viability assays

Infection of A549 cells was carried out in 60 mm dishes at a MOI of 10 PFU/cell. At 24, 48, 72, 96, and 120 h pi, medium was collected from samples and transferred to tubes in order to collect cells already detached from the monolayer. Adherent cells were trypsinized and pooled with collected medium. Pooled samples were then mixed with an equal volume of 0.4% Trypan Blue solution (Sigma, St. Louis, MO) and approximately 200 cells were counted using a light microscope and a hemocytometer. The percentage of viable cells was determined by dividing the number of unstained cells by the total number of cells counted and multiplying by 100.

### Cell viability of HeLa TREx cells overexpressing ORF E3-10.9K-EGFP fusion proteins

Previously generated plasmids pcDNA 4/TO EGFP, pcDNA 4/TO 4.8K-EGFP, pcDNA 4/TO 7.7K-EGFP, pcDNA 4/TO 9K-EGFP, and pcDNA 4/TO 10.9K-EGFP [21] were used to transfect low passage HeLa TREx cells (Invitrogen, Carlsbad, CA) using Effectene Reagent (Qiagen, Valencia, CA). Cells were maintained under 200 μg/mL Zeocin for two passages to select clones, and then maintained under 100 μg/mL Zeocin thereafter. Positive clones with efficient control of fusion protein expression were identified by their high expression of EGFP fusion protein upon the addition of 1 μg/mL tetracycline and used in subsequent experiments. To test cell viability of HeLa TREx cells expressing ORF E3-10.9K-EGFP orthologous fusion proteins, cells were plated at approximately 50% confluency in 6-well culture plates. One day post-plating, medium was aspirated and replaced with growth medium or growth medium supplemented with 1 μg/mL tetracycline. Medium was then collected and cells were trypsinized at 48 h post induction. Medium and cells were pooled and stained with an equal volume of 0.4% Trypan Blue solution (Sigma, St. Louis, MO). Approximately 200 cells were counted with a light microscope and a hemocytometer. Percent cell viability was determined by dividing the number of unstained cells by the total number of cells counted and multiplying by 100. Expression of EGFP fusion proteins was confirmed by live-cell fluorescence microscopy.

As a positive control for trypan blue staining of non-viable cells, HeLa TREx cells were plated in 60 mm culture dishes at approximately 50% confluency and harvested by trypsinization 48 h post plating. Cells were pelleted by centrifugation at 300 x g for 2 min and resuspended in 1 mL PBS. Three mL of cold (−20°C) absolute ethanol was slowly added to the cell suspension while gently mixing and incubated at −20°C for 15 min. Cells were then pelleted by centrifugation at 300 x g for 2 min and resuspended in 3 mL of PBS. Ethanol-treated cells were then stained with trypan blue solution and counted as indicated above.

## Competing interest

The authors declare that they have no competing interests.

## Authors’ contribution

The three authors participated in the design of the study and preparation of the manuscript. KMF carried out the entire body of experimental work presented in this paper. SKC constructed shuttle vector pE3-9K. AEK carried out sequence data analysis and annotation and was responsible for manuscript editing and submission.

## Supplementary Material

Additional file 1 ** Figure S1.** Viability of HeLa T-REx cells upon expression of EGFP-tagged ORF E3-10.9K orthologs. HeLa T-REx cells stably-transfected with pcDNA 4/TO expression plasmids were treated with 1 μg/mL tetracycline (+Tet) or mock-treated (−Tet). Prior to trypan blue staining, expression of EGFP-fusion proteins in tetracycline-treated (+Tet) or untreated (−Tet) live cells was examined by bright field (BF) and fluorescence (EGFP) microscopy. Images are representative of three independent experiments. Click here for file

Additional file 2 ** Table S1.** Viability of HeLa TREx cells expressing ORF E3-10.9K-EGFP orthologous fusion proteins. Click here for file
