# Supplementary figures and images for: No evidence of a death-like function for species B1 human adenovirus type 3 E3-9K during A549 cell line infection
Source: BMC Res Notes. 2012 Aug 11;5:429. doi: 10.1186/1756-0500-5-429 (PMC3500273; doi:10.1186/1756-0500-5-429)

## Slide 1
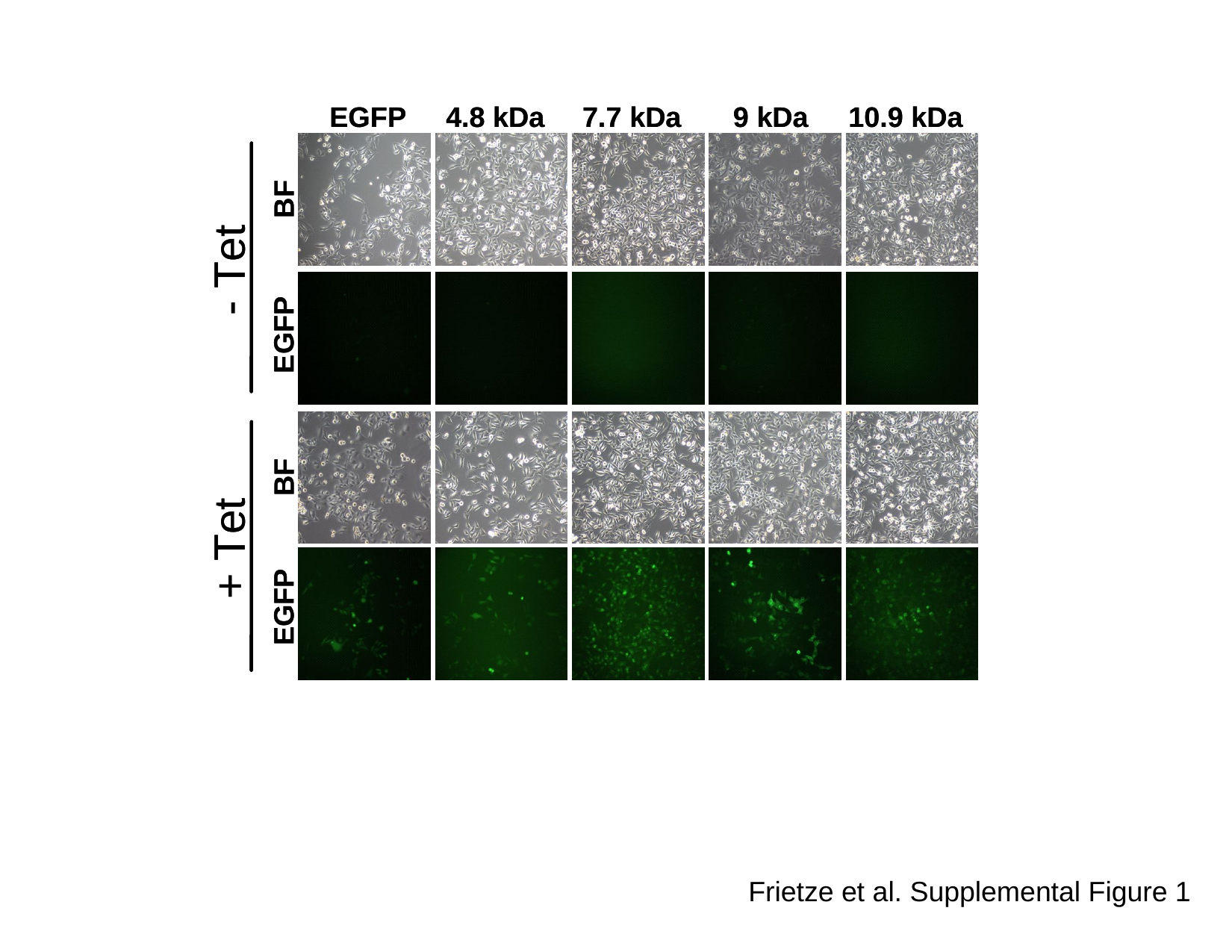

Frietze et al. Supplemental Figure 1

Supplement: Additional file 1 — Figure S1. Viability of HeLa T-REx cells upon expression of EGFP-tagged ORF E3-10.9K orthologs. HeLa T-REx cells stably-transfected with pcDNA 4/TO expression plasmids were treated with 1 μg/mL tetracycline (+Tet) or mock-treated (−Tet). Prior to trypan blue staining, expression of EGFP-fusion proteins in tetracycline-treated (+Tet) or untreated (−Tet) live cells was examined by bright field (BF) and fluorescence (EGFP) microscopy. Images are representative of three independent experiments. [file 1756-0500-5-429-S1.ppt]
