# Supplementary material for: No evidence of a death-like function for species B1 human adenovirus type 3 E3-9K during A549 cell line infection
Source: BMC Res Notes. 2012 Aug 11;5:429. doi: 10.1186/1756-0500-5-429 (PMC3500273; doi:10.1186/1756-0500-5-429)
Supplement: Additional file 2 — Table S1. Viability of HeLa TREx cells expressing ORF E3-10.9K-EGFP orthologous fusion proteins. [file 1756-0500-5-429-S2.docx]

**Supplemental Table 1:** Viability of HeLa TREx cells expressing ORF E3-10.9K-EGFP orthologous fusion proteins

|  | **% viable cells** | | | **Mean** | **SEM** |
| --- | --- | --- | --- | --- | --- |
| **Sample** | **Replicate 1** | **Replicate 2** | **Replicate 3** |  |  |
| **EGFP** | 96.6 | 94.8 | 96.0 | 95.80 | 0.92 |
| **E3-4.8K-EGFP** | 96.7 | 94.5 | 96.1 | 95.77 | 1.14 |
| **E3-7.7K-EGFP** | 98.5 | 91.5 | 94.1 | 94.70 | 3.54 |
| **E3-9K-EGFP** | 96.4 | 97.8 | 93.1 | 95.77 | 2.41 |
| **E3-10.9K-EGFP** | 98.4 | 96.4 | 97.2 | 97.33 | 1.01 |
| **Ethanol control** | 0 | 0 | 0 | 0 |  |
